# A Promising Vaccination Strategy against COVID-19 on the Horizon: Heterologous Immunization

**DOI:** 10.4014/jmb.2111.11026

**Published:** 2021-12-18

**Authors:** Sameer-ul-Salam Mattoo, Jinjong Myoung

**Affiliations:** Korea Zoonosis Research Institute, Department of Bioactive Material Science and Genetic Engineering Research Institute, Jeonbuk National University, Jeonju 54531, Republic of Korea

**Keywords:** SARS-CoV-2, variants of concern, heterologus vaccination, hybrid immunity

## Abstract

To overcome the ongoing COVID-19 pandemic, vaccination campaigns are the highest priority of majority of countries. Limited supply and worldwide disproportionate availability issues for the approved vaccines, together with concerns about rare side-effects have recently initiated the switch to heterologous vaccination, commonly known as mixing of vaccines. The COVID-19 vaccines are highly effective in the general population. However, none of the vaccines is 100% efficacious or effective, with variants posing more challenges, resulting in breakthrough cases. This review summarizes the current knowledge of immune responses to variants of concern (VOC) and breakthrough infections. Furthermore, we discuss the scope of heterologous vaccination and future strategies to tackle the COVID-19 pandemic, including fractionation of vaccine doses and alternative route of vaccination.

## Introduction

**Fig. 1:**
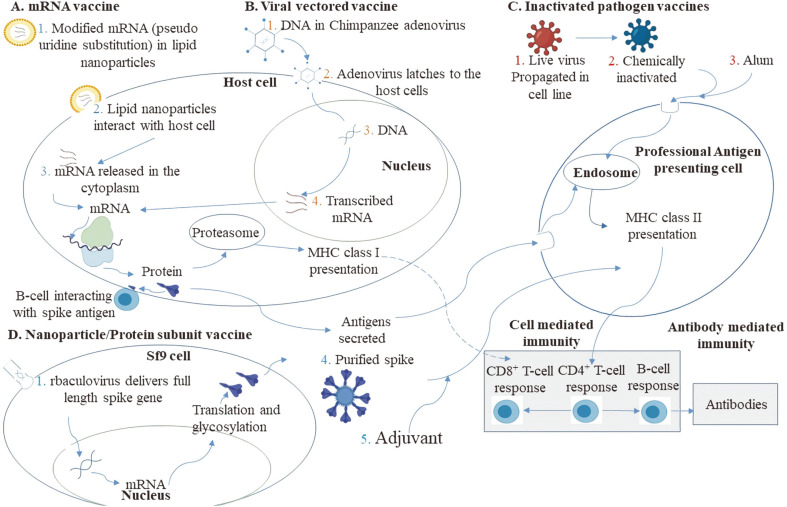
Proposed mechanism of action of COVID-19 vaccines. (**A**) In mRNA-vaccines, the spike mRNA is modified in which uridine is replaced by pseudouridine in order to escape immune responses. Moreover, the mRNA is stabilized in its prefusion conformation by two consecutive proline substitutions at amino acid positions 986 and 987, at the top of the central helix in the S2 subunit. mRNA is loaded in the lipid nanoparticles, which interact with the cell membrane and release modified mRNA in the cytoplasm of the muscle cell or antigen-presenting cell (1) (2) (3). mRNA is translated into spike protein in the cytoplasm, later presented by MHC class I to CD8^+^ T cells. The spike antigens are also released in the extracellular environment where they migrate to the draining lymph nodes and are endocytosed by APCs within the germinal centers. APCs at the site of injection may also be involved. These endocytosed antigens are processed by the presented by MHC class II to CD4^+^ T cells. Activated CD4^+^T cells help to activate CD8+T cells and B cells. CD8^+^ T cells kill the infected cells (not investigated in the context of SARS-CoV-2). With the help of CD4^+^ T cells, B cells mature to plasma secreting cells and synthesize antibodies to combat SARS-CoV-2. (**B**) In viral vectored vaccines, the full-length spike gene (DNA) is inserted in a harmless adenovirus vector (rAdenovirus) (1). rAdenovirus latches to the host cell and releases DNA in the cytoplasm (2) (3), which later migrates to the nucleus of the cell and is transcribed to mRNA (4). (**C**) Inactivated pathogen vaccines are chemically inactivated by β- propiolactone. The active component, along with the alum, generates immune responses. APCs process the antigens by MHC I machinery and present antigens to CD4^+^ T cells. (**D**) Full-length stabilized spike gene is engineered into baculovirus (rbaculovirus). rbaculovirus delivers spike gene into the Sf9 (1) (2), migrates to the nucleus of the cell, and is transcribed to mRNA. mRNA further migrates to the cytoplasm and is translated to spike protein (3). Translated and glycosylated spike protein is eventually purified and mixed with adjuvant (5). The dotted line represents that the exact role of CD8^+^ T cells is not known. MHC = major histocompatibility complex. mRNA = messenger RNA. r = recombinant. Sf9 cell = insect cell line, a clonal isolate derived from the parental *Spodoptera frugiperda* cell line IPLB-Sf-21-AE.

The coronavirus disease 2019 (COVID-19), caused by SARS-CoV-2, first emerged in China in December 2019. Since then, roughly 252 million cases of COVID-19 and 5.1 million deaths have been reported worldwide (as of November 12, 2021). Vaccines from different platforms are being used to overcome the pandemic [1]. Common ingredients of a vaccine include one or more active components and excipients, including lipids, adjuvants, buffer, salt, and sugar. The active component, also called an antigen, is the core of what makes vaccines work. An antigen is a substance foreign to the body that evokes an immune response either alone or after forming a complex with a larger molecule (such as a protein) and is capable of binding an antibody or T cell (Fig. 1).

The administration of the COVID-19 vaccines has resulted in a significant decrease in SARS-CoV-2 infections, hospitalization, and deaths associated with it [2-4]. However, in parallel to these outcomes, some rare adverse events have been at least temporally associated with vaccination. Due to limited vaccine administration in developmental and clinical phases, rare side effects emerge when millions of people are vaccinated during widespread use. Rare side effects, including vaccine-induced immune thrombotic thrombocytopenia (VITT), Guillain-Barré syndrome (GBS), and myocarditis, have been reported to be temporally associated with COVID-19 vaccines (reviewed in [5]). In addition, anaphylaxis (immediate-type reaction) and delayed-type hypersensitivity reactions, primarily supposed to be caused by excipients, have also been reported to be associated with COVID-19 vaccines. The concerns about rare side effects, VITT in particular, and more importantly the uneven availability issues for the emergency use approval (EUA) vaccines have incited policymakers to consider heterologous vaccination schedules.

None of the COVID-19 vaccine is 100% effective. Moreover, SARS-CoV-2 variant strains have emerged continuously. Some variants, especially VOC are highly transmissible and more resistant to immune responses. After complete immunization, the failure to mount or maintain effective immune responses against wild-type SARS-CoV-2 and, more importantly, against variants might lead to breakthrough cases (Table 1). A majority of the population is either partially vaccinated or unvaccinated. As of October 2021, 47.6% of the world's population has received at least one dose of a COVID-19 vaccine. In addition, only 2.7% of people in low-income countries have received at least one shot [6], which forebodes that the pandemic is far from over. Moreover, a limited supply of vaccines together with programmatic unfeasibility poses more hurdles to vaccinate the people.

This review provides a summary of immune responses to VOC and breakthrough cases. Furthermore, we discuss the potential of heterologous prime-boost vaccination and future strategies to tackle the COVID-19 pandemic, including fractionation of vaccine doses and alternative route of vaccination.

## Immune Responses to Variants of Concern 

Four variants of SARS-CoV-2 have been posing added threats to the ongoing pandemic: B.1.1.7 (Alpha), B.1.351 (Beta), P.1 (Gamma), and B.1.617.2 (Delta). A VOC is defined as a virus with mutations in multiple clusters in their genome with either detrimental changes in COVID-19 epidemiology, virulence, decreased effectiveness of public health measures, available diagnostics, vaccines, and/or therapeutics [7]. Compared to the Wuhan-1 reference strain (wild-type), VOC predominantly have mutations in the spike gene, altering their interactions with the host receptor ACE2, which results in higher infection rates. For example B.1.1.7 (~43-90%more transmissible compared with previous circulating strains) has H69/V70 and Y144 deletions; N501Y, A570D, D614G, and P681H substitution [8-10]. B.1.351 (25% more transmissible) has K417N, E484K, N501Y, and D614G key substitutions [10, 11]. P.1 (1.4–2.2 times more transmissible) has E484K, K417N/T, N501Y and D614G key substitutions [10, 12]. B.1.617.2 (97% more transmissible) has L452R, T478K, D614G, P681R key substitutions [11, 13]. VOC can also alter the potency of neutralizing antibodies, resulting in compromised vaccine efficacy and effectiveness. A properly timed and effective immune response is important for outsmarting the SARS-CoV-2. Firstly, a proper neutralizing antibody response would substantially decrease the number of virions that could successfully infect angiotensin converting enzyme-2 (ACE-2) receptor-expressing cells [14]. Neutralizing antibodies are able to bind to the virus and directly block its ability to infect cells, usually through inhibition of the interaction between the viral spike protein and the cellular ACE2 receptor [15]. Secondly, T cells might be playing a role in combating SARS-CoV-2 [15]. SARS-CoV-2-specific CD4^+^ T cells commonly differentiate into Th1 and Tfh T cells [16-18]. Th1 cells have antiviral properties, and Tfh cells are specialized in providing help to B cells and are critical for the development of neutralizing antibodies, memory B cells, and long-term humoral memory. Mostly Th1 skewed responses with little to none Th2 cytokines were detected in mRNA [19] and adenovirus vector-based vaccines [20] while Tfh cells have also been detected in vaccinated individuals [19, 21, 22]. In addition, CD8^+^ T cells can directly kill infected cells, which are also induced after SARS-CoV-2 infection or vaccination [16, 21]. The presence of virus-specific CD8^+^ T cells has been associated with better COVID-19 outcomes. SARS-COV-2-specific CD8^+^ T cells possess effector molecules, including IFN-γ, granzyme B, perforin, and CD107a [23-25]. In the case of inactivated COVID-19 vaccine, Th1 and Th2 T cell subsets were not defined although cellular immune responses in vaccinated individuals exhibited antigen-specific CD4^+^ and CD8^+^ T cells [26]. However, the role of T cells, especially CD8^+^ T cells, against SARS-CoV-2 remains to be elucidated. Although natural infection with SARS-CoV-2 and different vaccines induce more or less protective immunity, the ability of such immune responses to recognize and provide protection against variants of SARS-CoV-2 is a matter of concern.

### Antibody Responses

Planas *et al*. [27], evaluated the neutralizing potential of serum from BNT162b2, or ChAdOx1 nCoV-19 vaccinated individuals against D614G, B.1.1.7, B.1.351, and B.1.617.2 strains. After a single dose (post-3-weeks) of BNT162b2 vaccine, the levels of neutralizing antibodies were low against D614G and almost undetectable against the Alpha, Beta, and Delta variants. When evaluated 5-weeks after the booster, antibody titers significantly increased. However, in contrast to Alpha, 3- and 16-fold reductions in the neutralization titers against the Delta and the Beta variants, respectively, were observed. A similar pattern was observed with the ChAdOx1 nCoV-19. A single dose induced low levels of antibodies neutralizing the Delta and Beta variants (post-10-weeks) compared to the D614G and Alpha. Four weeks after the second dose, neutralizing titers were strongly increased. However, relative to the Alpha, 5- and 9-fold reductions in neutralization titers against the Delta and the Beta variants were observed. Further studies reported that BNT162b2 vaccinated individuals displayed 3.3-, 7.6-, 2.6-, and 2.5-fold reductions in the neutralization titer against Alpha, Beta, Gamma, and Delta variants, respectively, in contrast to Victoria strain [28-31]. Moreover, ChAdOx1 nCoV-19 vaccinated individuals showed a 2.33-, 9-, 2.9-, and 4.29-fold loss in neutralization titer against Alpha, Beta, Gamma, and Delta variants, respectively, compared with Victoria [28-31]. In the following sections vaccination refers to full vaccination as recommended by respective manufacturers unless otherwise mentioned.

**B.1.1.7 VOC.** The sera from mRNA or viral vectored vaccinated individuals showed a small yet significant reduction (1.7 to 2.5-fold) in neutralizing activity against B.1.1.7 compared to the reference strains [28, 32-37]. However, a 9-fold loss in neutralization potential of serum from ChAdOx1 nCoV-19 vaccinated individuals was also reported. Serum from inactivated pathogen vaccine, CoronaVac, vaccinated individuals showed a 17.35-fold loss in geometric mean titer of neutralizing antibodies against the authentic virus, compared with wild-type SARS-CoV-2 [38]. Other studies suggest that neutralizing activity of mRNA or viral vectored vaccinated individuals sera likely maintains protective efficacy against B.1.1.7 [30, 39, 40]. All these in-vitro studies have certain limitations and variations in methodology, sample size, sampling time, and considering only the humoral arm of the immune response (Table 2). For example, volunteers in phase 2/3 vaccine efficacy study of ChAdOx1 nCoV-19 showed a 9-fold reduced serum neutralization activity against B.1.1.7 in comparison to a canonical non-B.1.1.7 Victoria strain [37]. In contrast, the vaccine was 70.4% effective against nucleic acid amplification test of nasal swabs for B.1.1.7 (81.5% effective against non- B.1.17 lineages).

**P.1 VOC.** Serum neutralization assay using a pseudovirus system deciphered, 1.2 to 5.12-fold reductions in neutralization against P.1 for mRNA vaccinees serum [32, 33, 41]. Serum from Ad26.CoV.S vaccinees showed a 3.3-fold reduction in neutralization potential compared with WA1/2020 [42]. Geometric mean serum neutralization titers against P.1 were reduced by 2.6-fold for the BNT162b2 and 2.9-fold for the ChAdOx1 nCoV-19 vaccinee’s serum against authentic virus, relative to the Victoria strain [30].

**B.1.351 VOC.** In contrast to Alpha, a 9 to 16-fold reduction in the neutralization titers of serum from BNT162b2 vaccinees against the Beta variant has been observed. The neutralization potential of sera from BNT162b2 (1 to 5-weeks post-second dose) was 7.6 to 16-fold resistant to B.1.351 as compared with reference strains [27, 29, 30, 34, 39, 40, 43, 44]. Likewise, the neutralization potential of sera from mRNA-1273 vaccinees was 5 to 12.4-fold lesser, compared with reference strains [33, 35, 40]. Serum from ChAdOx1 nCoV-19 (4-weeks post-second shot) or Ad26.CoV.S (71 days post-vaccination) vaccinees showed 9- and 10.6-fold reductions in neutralization potential compared with B.1.1.7 and WA1/2020, respectively [27, 42]. Another study evaluated serum from CoronaVac vaccinees, reported a 22.11-fold loss in geometric mean titer of neutralizing antibodies against B.1.351, compared with wild-type SARS-CoV-2 [38].

In a phase III clinical trial, a single shot of Ad26.COV2.S has shown 64.0% and 80% efficacy for moderate to severe–critical COVID-19 against the B.1.351 and B.1.1.7 variants (96% efficacy against the original strain). Despite reduced neutralizing antibody titer (>10.6-fold lower against B.1.351 compared with WA1/2020), the protective efficacy of Ad26.COV2.S might be due to CD8^+^ T cells and functional non-neutralizing antibodies [42].

**B.1.617.2 VOC**. Post BNT162b2 vaccination, the sera from individuals showed a 1.14 to 5.8-fold reduction in neutralization titer against B.1617.2 compared with reference strains [34, 45]. The serum from mRNA-1273 vaccinated individuals showed a 2.1-fold reduction compared with D614G [35]. Edra *et al*. [46] reported a 3.3-fold and 3-fold decrease in the neutralizing antibody titer in the serum of BNT162b2 and mRNA-1273 vaccinated individuals compared with the WA1/2020 SARS-CoV-2. Liu *et al*. [31], showed 2.5-fold and 4.29-fold reductions in the neutralizing antibody titers against B.1.617.2 in the serum of BNT162b2 or ChAdOx1 nCoV-19 vaccinated individuals, respectively, compared with Victoria strain. Compared with wild-type SARS-CoV-2, a 31.64-fold loss in geometric mean titer of neutralizing antibodies against authentic B.1.617.2 has been reported in CoronaVac vaccinated individuals [38].

Although the VOC more or less escape neutralization by antibodies and there are reports of infection by variants in the vaccinated population, the vaccines effectively reduce the severity of the disease (Table 1). Currently, the prevention of severe disease and deaths is of utmost importance. However, the resilience of immune responses elicited by COVID-19 vaccines, especially against VOC, remains to be elucidated. Although, an 8-month study in which 20 participants received the Ad26.COV2.S vaccine in 1 or 2 doses (either 5×10^10^ viral particles or 10^11^ viral particles), reported durable humoral and cellular immune responses with expanding neutralizing antibody breadth against variants [47]. Individuals receiving a single-shot regimen had a median pseudovirus-neutralizing antibody titer of 272 and 184 against the parental WA1/2020 strain, 167 and 158 against the D614G, 60 and 147 against the B.1.1.7, 39, 107 against the B.1.617.2, 28 and 129 against the P.1, <20 and 62 against the B.1.351 on days 29 and 239, respectively. However, this study has its limitations of including low sample size, use of pseudovirus assay instead of authentic live virus, lack of comparison between different dose regimes, and lack of evaluation of memory B and T cells.

Summary of different methodologies used to evaluate the antibody responses in vaccinated indididuals has been provided in Table 2.

### T Cell Responses

Currently, most of the vaccines contain spike [19, 48, 49], and mutations have been widely reported in the spike. Antibodies induced by spike of the prototypic strain of SARS-CoV-2 have less binding and neutralization abilities for newly emerging variants resulting in escape from the antibody responses. SARS-CoV-2 antibody responses have received a lot of attention. However, the arsenals of humoral and T cell responses may play diverse roles in different viral infections. In addition, T cells induced by vaccines are supposed to recognize SARS-CoV-2 variants [50, 51]. For example, a study evaluated WT and variants of SARS-CoV-2 specific CD4^+^ and CD8^+^ T cell responses in BNT162b2 (*n* = 8) or mRNA-1273 (*n* = 11) vaccinees (samples were collected 2-4 weeks after the second dose of vaccination) [50]. Peptide mega pools (MPs) spanning the entire SARS-CoV-2 proteins or only spike were used to stimulate PBMCs, and the response was evaluated based on activation-induced markers (AIM) in CD4^+^ (OX40^+^ CD137^+^) and CD8^+^ (CD69^+^ CD137^+^) T cells. The CD4^+^/CD8^+^ T cell reactivity in the vaccinees was not substantially affected by mutations in B.1.1.7 and P.1. However, decreases of 14% and 22% were observed with the B.1.351 spike-pools for CD4^+^ and CD8^+^ T cells, respectively. AIM T cell responses in COVID-19 vaccinees displayed a memory phenotype irrespective of the variant analyzed, with preferential enrichment for central memory (T_cm_) and effector memory (T_em_) for CD4^+^ and T_em_ and terminally differentiated effector memory (T_emra_) for CD8^+^ T cells. These provide evidence that donors primed by the ancestral strain spike protein mount a memory T cell response that can cross-recognize the SARS-CoV-2 VOC. A limitation of this study is that overlapping peptide pools rather than individual peptides were used to evaluate the responses by which alterations in terms of antigen processing for either class I or class II MHC would be undetected. In another study, peptide pool (15-mers with 11 amino acids overlap) spanning mutated spike regions of B.1.1.7 and B.1.351 were used to detect the cross-reactivity of SARS-CoV-2 specific T cells with variants [51]. Blood samples were collected from COVID-19 naïve and recovered donors before and after the BNT162b2 vaccination. No differences in CD4^+^ T cell activation (based on AIM) were seen in response to variant antigens. However, in this study, the number of donors was limited to 20, and CD8^+^ T cells responses to VOC were not evaluated. In contrast, a study evaluated 747 SARS-CoV-2 virus isolates by deep sequencing and reported that MHC-I restricted mutant epitopes showed reduced (assessed based on melting temperature stabilizing capacity of wildtype or mutant peptides towards MHC-I) or even abrogated (HLA tetramers, loaded with WT or mutant peptide, were presented to expanded CD8^+^ T cells of HLA-matched COVID-19 patients) binding to MHC-I [52]. Moreover, CD8^+^ T cells stimulated with respective epitopes showed decreased proliferation and cytotoxicity. The tetramer-sorted CD8^+^ T cells revealed qualitative differences at the transcriptional level to mutant peptides. However, this approach should be extended to evaluate the T cell response after vaccination/immunization.

## Breakthrough Cases

Breakthrough cases are people who get an infection even after complete immunization, meaning the pathogen breaks the protective barrier developed by vaccination. As already stated, none of the COVID-19 vaccines is 100%effective. Moreover, SARS-CoV-2 variant strains have emerged continuously. Reduced antibody responses in susceptible populations might render them prone to breakthrough infections [53]. Moreover, VOC may escape immune responses, so breakthrough cases are expected (Table 1). For example, a study including 1497 fully vaccinated health care workers reported 39 SARS-CoV-2 breakthrough infections. For 22 of the 39 workers with breakthrough infections, the results for peri-infection neutralizing antibodies were available. During the peri-infection period, the neutralizing antibody titers in breakthrough cases were lower than those in matched uninfected vaccinated controls (*n* = 104) [54]. Although higher peri-infection neutralizing antibody titers were associated with lower infectivity, the levels of neutralizing antibodies in breakthrough cases were not significantly lower than matched uninfected vaccinated controls. Moreover, this analysis does not provide a specific level of antibodies that might be associated with protection [55]. Another study reported lower levels of antibodies (S-RBD IgG, 3.469 arbitrary units /ml, AU/ml) in a 41-year-old woman 34-days post complete vaccination [56] compared to a previous study [57]. This patient developed COVID-19 symptoms 40-days post-vaccination. Subsequently, 20-days post-symptom onset, the titer of the spike protein receptor-binding domain (S-RBD) IgG antibodies increased to 130 AU/ml. These results show that the vaccine failed to develop an effective immune response in the patient. In contrast, Hacisuleyman *et al*.[58] reported 2 breakthrough cases among 417 mRNA vaccinated individuals (19 and 36 days post-complete vaccination) [58]. One patient had extremely high titers of neutralizing antibodies. Moreover, the antibodies recognized the variants but were nonetheless insufficient to prevent a breakthrough infection. However, it can’t be ruled out that the infection may have occurred before the booster shot took full effect.

## Heterologous Vaccination

### Background

In March 2021, vaccinations with ChAdOx1 nCoV-19 were abruptly halted due to VITT [59, 60]. The activation of platelet factor 4 (PF4) by antibodies might be amplified by booster vaccination with an adenoviral vector, which might induce and/or aggravate its adverse reactions. In addition, immune responses to the viral vector itself might compromise vaccine efficacy. Thus, boosting with an mRNA-based vaccine have instead been recommended [61]. Moreover, uneven availability issues for the approved vaccines around the world also compelled the switch to heterologous vaccination schedules [62]. A heterologous prime-boost vaccination (HtPBV) strategy could be an opportunity to make vaccination programs more flexible and reliable in response to fluctuations in supply or demand [61]. However, HtPBV has also been evaluated before COVID-19, and in many scenarios, heterologous vaccination has been more immunogenic than homologous prime-boost vaccination (HmPBV) [63, 64]. In the context of COVID-19, some initial reports demonstrate that HtPBV is better or at least as immunogenic as HmPBV (Table 3).

### Safety and Efficacy

Com-COV is a participant-blinded, randomized, phase 2, UK multicenter, non-inferiority study investigating the safety, reactogenicity, and immunogenicity of HtPBV COVID-19 vaccine schedules (interval between first and second shot = 28 days). As per the initial reactogenicity data, both heterologous vaccine schedules (ChAdOx1 nCoV-19–BNT162b2 prime-boost and BNT162b2–ChAdOx1 nCoV-19 prime-boost) induced greater systemic reactogenicity following the boost shot than their homologous counterparts (ChAdOx1 nCoV-19 prime-boost and BNT162b2-prime-boost [65]. In this study, up to 80% of individuals receiving a HtPBV reported fatigue and other systemic reactions, an up to 40 times increase compared with the HmPBV. Feverishness was reported by 37 (34%) of 110 recipients of ChAdOx1 nCoV-19 for prime and BNT for boost compared with 11 (10%) of 112 recipients of ChAdOx1 nCoV-19 for both prime and boost. In addition, feverishness was also reported by 47 (41%) of 114 recipients of BNT for prime and ChAdOx1 nCoV-19 for boost, compared with 24 (21%) of 112 recipients of BNT for both prime and boost (difference 21%, 95% CI 8-33%). Similar increases were observed for chills, fatigue, headache, joint pain, malaise, and muscle ache. Most of this increase in reactogenicity was observed in 48 h after immunization. However, there were no hospitalizations due to solicited symptoms. In contrast, a prospective observational cohort study demonstrated no major differences in reactogenicity between the prime-boost regimens [66]. Between December 27, 2020, and June 14, 2021, 380 participants (median age = 35, wome*n* = 62%) were enrolled in this study, with 174 receiving BNT162b2 HmPBV vaccination, 38 receiving ChAdOx1 nCov-19 prime-boost homologous vaccination vaccination, and 104 receiving ChAdOx1 nCoV-19–BNT162b2 HtPBV vaccination (interval between first and second dose = 71 days for ChAdOx1 nCoV-19–BNT162b2 and median 83 days for homologous ChAdOx1 nCov-19). Systemic symptoms were reported by 103 (65%) of 159 recipients of homologous BNT162b2, 14 (39%) of 36 recipients of homologous ChAdOx1 nCov-19, and 51 (49%) of 104 recipients of ChAdOx1 nCov-19–BNT162b2 after the booster immunization. Local reactions were frequently observed for all vaccination regimes. Systemic reactions, including severe reactions, were most frequent after prime immunization with ChAdOx1 nCoV-19. Reactogenicity of HmPBV (BNT162b2–BNT162b2), HmPBV (ChAdOx1 nCoV19–ChAdOx1 nCoV19), and HtPBV (ChAdOx1 nCoV19–BNT162b2) were similar, with slightly decreased systemic reactions after HtPBV (ChAdOx1 nCov-19–BNT162b2) and HmPBV (ChAdOx1 nCov-19–ChAdOx1 nCoV19). The difference in study design, population demographics, and prime-boost vaccination interval might be responsible for the discrepancy in these two studies.

HtPBV induces effective humoral and cellular immune responses in vaccinees (Table 3). One of the initial studies compared ChAdOx1 nCoV-19, BNT162b2 HtPBV with ChAdOx1 nCoV-19 prime and no boost vaccinated groups [61]. RBD antibody-titer, trimeric spike protein antibody titers, and neutralizing antibodies were significantly higher in HtPBV than ChAdOx1 nCoV-19 primed group. Moreover, overnight stimulated whole blood with pools of SARS-CoV-2 spike peptides displayed significantly higher INF-γ levels in HtPBV than ChAdOx1 nCoV-19 primed group. A prospective cohort study evaluated BNT162b2, ChAdOx1 nCoV-19 HmPBV and BNT162b2, ChAdOx1 nCoV-19 HtPBV and deciphered an increased spike S1-reactive T cell responses in HtPBV [66]. The geometric means of 50% inhibitory dose against Alpha and Beta variants were highest in recipients of ChAdOx1 nCov-19 BNT162b2 HtPBV compared with the recipients of ChAdOx1 nCov-19 or BNT162b2 HmPBV. Next study evaluated ChAdOx1 nCoV-19–ChAdOx1 nCoV-19 HmPBV with ChAdOx1 nCoV-19–BNT162b2 HtPBV [67]. In contrast to HmPBV, HtPBV significantly induced higher frequencies of spike specific CD4^+^ and CD8^+^ T cells and in particular, induced high titers of neutralizing antibodies against VOC (B.1.1.7 and B.1.351, and P.1). Schmidt *et al*. [68] reported a significantly higher frequency of activated CD69^+^IFN-γ^+^CD8^+^ T cells in HtPBV than HmPBV. A single case report deciphered that ChAdOx1 nCoV-19–BNT162b2 HtPBV elicited a robust humoral immune response [69], exceeding the levels reported by Mulligan *et al*. [70] in BNT162b2–BNT162b2 HmPBV. Other studies reported a comparable immune responses in BNT162b2–BNT162b2 HmPBV and ChAdOx1 nCoV-19–BNT162b2 HtPBV [63, 71]. Overall, it seems that HtPBV elicits at least comparable or even better immune responses. In the context of heterologous vaccination, Com-COV study will recruit more individuals to evaluate mRNA-1273 and NVX-CoV2373 mixing [64]. HtPBV will at least help to counterbalance the shortage of one or more vaccines. However, the durability of such a regime to maintain protection over longer periods should be evaluated. Moreover, the efficacy and effectiveness of HtPBV against variants should be given more attention.

### Hybrid Vigor Immunity

Immunological memory induced by vaccines is a source of protection against infection. However, the vaccine effectiveness is more or less reduced against VOC [2-4, 72]. On the other hand, natural infection by SARS-CoV-2 also induces memory immune responses. However, reinfections, especially with variants, including B.1.315 have been reported. What happens when previously infected individuals are vaccinated? The reports from several studies suggest that an impressive synergy results from a combination of natural immunity and vaccine-generated immunity called “hybrid vigor immunity” [73]. Natural immunity to SARS-CoV-1 or SARS-CoV-2, combined with vaccine-generated immunity, generates broad immune responses. For example, Tan *et al*. [74] investigated the possibility of a cross-clade boost of broad-spectrum neutralizing antibodies in survivors of SARS-CoV-1 infection in Singapore who had received the BNT162b2 [74] (124). They assessed the immune responses to the BNT162b2 in the survivors of SARS-CoV-1 infection (*n* = 10), survivors of SARS-CoV-2 infection (n =10), and uninfected individuals (*n* = 10). After receiving two doses of the BNT162b2 vaccine, the SARS-CoV-1 survivors had neutralizing antibodies against 10 different sarbecoviruses virus – 7 from SARS-CoV-2 clade viruses (the original strain of SARS-CoV-2; SARS-CoV-2 VOC B.1.1.7, B.1.351, B.1.617.2; bat coronavirus RaTG131; and pangolin coronaviruses GD-112 and GX-P5L12) and 3 from SARS-CoV-1 clade (SARS-CoV-1, bat WIV1,13, and bat RsSHC01413). SARS-CoV-2 survivors and healthy vaccine recipients had neutralizing antibodies to SARS-CoV-2 clade isolates, but significantly lower levels of neutralizing antibodies against SARS-CoV-1 clade. Stamatatos *et al*. [75] evaluated sera from 15 individuals who had previously been infected with SARS-CoV-2 and 13 individuals who had not been infected. The sera were collected before and after immunization with one of the mRNA vaccines (BNT162b2 or mRNA-1273). Prior to vaccination, sera from 12 of the 15 previously infected donors neutralized the Wuhan-Hu-1. However, the sera from these individuals showed weak and only sporadic neutralizing activity against the B.1.351. Interestingly, a single shot of vaccine in previously infected individuals with pre-existing virus-specific antibodies induced higher levels of virus-specific IgG and IgA than two vaccine doses in naive individuals. Compared to two vaccine doses in naïve individuals, a single dose of vaccine in previously infected individuals displayed 10- and 20-fold higher levels of neutralizing antibodies to the Wuhan-Hu-1 and B.1.351, respectively. Nevertheless, the serum of previously infected vaccinated individuals was 3 to 10-fold less efficient in neutralizing the B.1.351 compared with Wuhan-Hu-1. Moreover, a second dose of the vaccine in the previously infected individuals within 3-4 weeks did not further boost neutralizing antibodies levels. Goel *et al*. [76] evaluated antibody and antigen-specific memory B cells in 33 SARS-CoV-2 naïve and 11 SARS-CoV-2 recovered subjects. Both groups received SARS-CoV-2 mRNA vaccines (BNT162b2 or mRNA-1273). SARS-CoV-2 naïve individuals required both vaccine doses for optimal increases in antibodies. Memory B cells specific for full-length spike protein and the RBD were also efficiently primed by mRNA vaccination and detectable in all SARS-CoV-2 naive subjects after the second vaccine dose. In SARS-CoV-2 recovered individuals, antibody and memory B cell responses were significantly boosted after the first vaccine dose. However, there was no increase in circulating antibodies, neutralizing titers, or antigen-specific memory B cells after the second dose. This robust boosting after the first vaccine dose strongly correlated with levels of pre-existing memory B cells in recovered individuals, identifying a key role for memory B cells in mounting recall responses to SARS-CoV-2 antigens.

In summary, hybrid vigor immunity is a potential field to explore the active components of COVID-19 vaccines. It is interesting to note that currently available vaccines mostly employ the spike protein as immunogen. Including other viral genome components alongwith the spike in COVID-19 vaccines may mimic the natural virus more closely. And more importantly, development of replication-defective vaccines using the reverse genetics might pave way to better vaccines by inducing and mimicking the hybrid immunity described above. Idenfifying and deletion of viral factors [77-85], which modulate the host interferfon reponses, need to be considered for the development of next-generation COVID-19 vaccines.

## Future Strategies: Fractional Dosing of Vaccines and Route of Vaccine Administration

In the context of COVID-19, various public health and social measures have been implemented to control the transmission of SARS-CoV-2. However, being emergency measures, they are difficult to sustain for longer periods [86]. Besides, a shortage in the supply of vaccines is a matter of concern, especially in low-income countries. However, if dose-sparing is effective in preventing symptomatic and severe disease, it would extend the limited supply of vaccines and will play a significant role in bringing the pandemic to an end. More importantly, vaccinating more people with lesser doses may reduce the transmission of the virus, which might reduce the incidence and occurrence of the disease [86]. Dose sparing in case of COVID-19 vaccines shall be evaluated to answer a number of questions [87]: Will dose sparing result in an abundant immune response to prevent symptomatic or severe disease and transmission of the virus; how effective will it be against VOC; how safe will it be to administer, including adverse reactions and emergence of new variants; will it be effective in different populations, including immunocompromised individuals? A primising example of successful vaccine dose fractionation is against yellow fever in Angola, the Democratic Republic of Congo. In 2015, in response to the yellow fever epidemic, emergency vaccination was required. However, due to the limited supply of vaccines, WHO’s Strategic Advisory Group of Experts on Immunization reviewed the evidence on the immunogenicity and safety of fractional dosing of vaccines against yellow fever and recommended dose fractionation down to one-fifth of the standard dose [88, 89]. Fractional dosing was predicted to substantially reduce population infection attack rates and save lives [88]. In the context of COVID-19 vaccines, a preliminary study comprising 600 individuals of different age groups evaluated 50 and 100 μg 2-dose regime (mRNA-1273) for safety and immunogenicity [90]. Anti-SARS-CoV-2 spike binding antibody levels increased substantially by day 14 after the second dose to geometric mean peak levels of 189 (173-207) and 239 (221-259) μg/ml at 50 and 100 μg dose respectively in younger participants (≥18 to <55-years age), and 153 (135-175) and 162 (142-185) μg/ml in older participants (≥55 years age). In addition, neutralizing antibody levels were increased to maximum geometric mean titers of 1733 (1611-1865) μg/ml at 50 μg dose and 1909 (1849-1971) μg/ml at 100 μg dose in younger adults, and 1827 (1722-1938) μg/ml at 50 μg and 1686 (1521-1869) μg/ml at 100 μg in older adults. Although no statistical evaluation was done for antibody levels in participants who received 50 or 100 μg doses, numerical antibody levels seem to be comparable which favors the feasibility of fractional dosing [90]. In an interim analysis of 4 randomized controlled trials, a subgroup of participants was primed with a half dose of ChAdOx1 nCoV-19 vaccine instead of a full dose, followed by a full-dose boost after a median of 12 weeks [91]. A vaccine efficacy of 90% (67-97%) was reported in this subgroup. Although only a small number of participants were included, the lower bound of 67%for the efficacy estimate is very reassuring [86]. However, fractional dosing of COVID-19 vaccines needs to be evaluated in larger populations especially because immune correlates of protection have not been established.

In the UK, a decision was made in December 2020 to delay the second vaccine dose to 12-weeks post-first dose, which aimed to vaccinate more people to develope at least some protection against SARS-CoV-2. A third wave of COVID-19 caused by a highly transmissible Delta variant has led to considerations of the potential need and optimal timing for a second booster shot for vaccinated populations [92]. However, vaccinating more people appears more tempting. Two doses of COVID-19 vaccines are efficient in controlling severe disease, even those caused by VOC [2-4]. Although there are concerns about waning antibody responses, however, the declining antibody responses do not necessarily mean reduced vaccine efficacy because the effect against disease is not only mediated by antibodies that might be relatively short-lived for some vaccines but also by long-living memory and cellular immune responses [93]. For influenza, each annual vaccine is based on the most current data about circulating strains, increasing the likelihood that the vaccine will remain effective even if there is further strain evolution [94]. In the sense of COVID-19, there is an opportunity now to study variant-based boosters before there is a widespread need for them [95]. In this context, Moderna has started clinical trials (NCT04785144) for mRNA-1273.351, targeting novel B.1.351 VOC. The study is divided into 2 cohorts. Cohort 1 who received two vaccinations of mRNA-1273 at dosages of 50 μg, 100 μg, or 250 μg in the Phase 1 clinical trial (DMID 20-0003) will be given a single intramuscular (IM) booster of mRNA-1273.351. Cohort 2, who have never received a COVID-19 vaccine, will be given 2 or 3 IM doses of mRNA-1273.351. Moreover, a multivalent booster candidate mRNA-1273.211 (Combines mRNA-1273 and mRNA-B.1.351) to adult participants who previously received 2 doses of mRNA-1273 (NCT04470427) is currently in Phase 2 and 3 (NCT04927065).

SARS-CoV-2 specific T cells have been detected even in asymptomatic individuals [96] and those who don’t seroconvert [97]. T cells can be especially important in convalescents who don’t seroconvert or immunocompromised individuals who are less likely to develop an effective antibody response. Sterilizing immunity completely stops viral replication in the host, which can be achieved by antibodies. Among T cells, CD8^+^ T_RM_ could come closest to sterilizing immunity by eliminating the pathogens at the portal of entry [98]. The route of COVID-19 vaccine administration shall be given more attention as both route and vaccine formulation are key determinants for T_RM_ formation [99, 100]. For example, the parenteral route of administration is unable to efficiently induce IgA and T_RM_ in the lungs [101, 102] in comparison to intranasal (IN) vaccination. A single IN dose of Chimpanzee adenoviral vaccine encoding stabilized S in mice almost entirely prevented SARS-CoV-2 infection in both the upper and lower respiratory tracts by inducing a mucosal immune response, including high levels of SARS-CoV-2 S specific IgA in serum and lung. Of note, CD103^+^CD69^+^CD8^+^ T cells, likely of a resident memory phenotype, were induced by IN route and not by the IM route [102]. These results depict that intramuscular vaccination does not confer sterilizing immunity. Eventually, Hassan *et al*. [102] extended their strategy to non-human primates and found that a single dose of IN adenoviral vectored vaccine protects rhesus macaques against SARS-CoV-2. However, in this study, IM and IN routes were not compared. Currently, 7 vaccines are in clinical phase trials which will be administrated by IN route. However, how effective IN vaccination will be, primarily in the long run, need to be evaluated in a more controlled and strict manner. A typical exemplary to understand the immune kinetics of IN immunization is vaccination against Influenza A virus (IAV) [reviewed by [103]. IAV specific lung T_RM_ provides potent protection against heterosubtypic influenza challenge. However, this protection is transient because of increased apoptosis of T_RM_ in the lung and airways, unlike populations in the skin, nasal tissue, and intestinal mucosae. In this regard, COVID-19 vaccines effectively inducing and stabilizing T_RM_ in the lungs will be an exciting field to explore.

In conclusion, VOC, especially B.1.315 and B.1.617.2, escape the antibody responses. The failure to generate sufficient immune responses might lead to breakthrough cases. However, recommended doses of vaccines are effective against severe diseases and deaths that are of utmost importance in the present scenario.

The uneven availability of COVID-19 vaccines can be tackled by heterologous vaccination, which generates better or at least comparable immune responses. The reports about the adverse reactions of heterologous vaccination are rare and shall be evaluated in larger populations. An emerging concept of hybrid vigor immunity shall be given prime attention. In this context, the inclusion of different SARS-CoV-2 proteins along with spike may provide broader protection against SARS-CoV-2 variants.

## Figures and Tables

**Table 1:** Breakthrough cases.

Vaccination regime	Time of study or follow-up	Population size	Breakthrough cases	Time post second dose	Persistent infection or severe/critical/fatal/hospitalization	Prevalent/Identified SARS-CoV-2 strain (number of cases)	Reference
mRNA-1273	Feburary to May 2021	2520	6	≥ 14 days	1	B.1.1.7 B.1.351 (6)	[3]
BNT162b2	February 23 to March 18, 2021	50850	474	≥ 14 days	3 (B.1.351)	B.1.1.7 (51) B.1.351 (201) Unknown (222)	[4]
BNT162b2	4-month period after the second shot	1497	39	≤ 4-month	19%	B.1.1.7	[54]
BNT162b2 or mRNA-1273	January 21 to March 17, 2021, and weekly testing continued thereafter	417	2	≥ 14 days	__	__	[58]
BNT162b2 or mRNA-1273	March 9 to May 6, 2021	2380 (received at least 1 dose)	17	≥ 14 days	__	__	[104]
BNT162b2	January 23 to March 7, 2021	792	76	≥ 14 days	__	B.1.1.7 B.1.351	[105]
ChAdOx1	January 16 to May 21, 2021,	1322	108	14 days	6 required hospitalization	__	[106]
BNT162b2 or mRNA-1273	December 15, 2020 to March 30, 2021	258,716	271	≥ 14 days	42 had severe disease		[107]
BNT162b2	__	1137	4	62 days	prolonged viral shedding up to 32 days after diagnosis	B.1.1.7	[108]
BNT162b2, mRNA-1273, and Ad26.COV2.S	February–April 202	103,166 (BNT162b2) 20,345 (mRNA-1273) 2,856 (Ad26.COV2.S)	^a^101	≥14 days after last dose	^b^7 hospitalized, ^c^1 died	B.1.1.7 B.1.526	[109]
CoronaVac	July to December 2020	651	2	^d^106 and 122 days	^e^2	P.1	[110]
BNT162b2	March to April 2021	_	21	≥14 days	__	B.1.1.7.	[111]
BNT162b2	March 15 to May 6 2021	70	12	1-month	1	B.1.1.7	[112]

^a^Out of 101, 76 cases (75%) yielded full SARS-CoV2 genomes (61= BNT162b2, 11= mRNA-1273, and 4= Ad26.COV2.S). ^b^4 BNT162b2 vaccinated, 2 Ad26.COV2.S vaccinated and 1 mRNA-1273 vaccinated. ^c^An elderly patient with multiple comorbidities who already was on in-home oxygen previous to post-vaccination COVID-19 infection and had a lengthy stay at the ICU. ^d^diagnosis of breakthrough infection in patient 1 and patient 2, 106 and 122 days, respectively, following administration of the 2nd vaccine dose. ^e^One patient had history of diabetes mellitus type 2, high blood pressure, and obesity degree I.

**Table 2:** Antibody escape by SARS-CoV-2 variants.

Vaccination regime (n)	Sampling time post second dose	Assays for antibody titration	SARS-CoV-2 strain (fold reduction)	Reference
BNT162b2	5-weeks	S-Fuse neutralization assay using authentic virus (ED_50_)	B.1.1.7 (reference) B.1.351 (16) B.1.617.2 (3)	[27]
ChAdOx1 (23)	4-weeks		B.1.1.7 (reference) B.1.351 (9) B.1.617.2 (5)	
BNT162b2 (25)	7 to 17-days	Authentic virus (FRNT_50_)	Victoria (reference) B.1.1.7 (3.3)	[28]
ChAdOx1 (15 and 10)	14 and 28-days		Victoria (reference) B.1.1.7 (2.5-2.1)	
BNT162b2 (25)	7 to 17-days	Authentic virus (FRNT_50_)	Victoria (reference) B.1.351 (7.6)	[29]
ChAdOx1 (25)	14 and 28-days		Victoria (reference) B.1.351 (9)	
BNT162b214 (25)	7 to 17-days	Authentic virus (FRNT_50_)	Victoria (reference) P.1 (2.6)	[30]
ChAdOx1 (25)	14 and 28		Victoria (reference) P.1 (2.9)	
BNT162b2 (25)	7 to 17-days	Authentic virus (FRNT_50_)	Victoria (refrence) B.1.617.2 (2.5)	[31]
ChAdOx1 (25)	14 or 28		Victoria (refrence) B.1.617.2 (4.29)	
BNT162b2 (24)	1-week	Authentic infectious virus (FRNT)	WA1/2020 (reference) B.1.1.7 (2)	[36]
BNT162b2 (15)	13 to 15-days	VSV pseudotyped with the S proteins of SARS-CoV-2 variants (NT_50_)	Wuhan-1 isolate with D614G exchange (reference) B.1.1.7 (1.77) B.1.351 (7.85) P.1 (5.12)	[32]
mRNA-1273 (8)	1-week	VSV pseudotyped with the S proteins of SARS-CoV-2 (ID_50_)	D614G (reference) B1.1.7(1.2) B.1.1.7+E484K (3.1) P1 (3.5) B.1.351 (6.4)	[33]
BNT162b2 (159)	28-days (mean)	Authentic virus (IC_50_)	hCoV19/England/02/2020 (reference) B.1.1.7 (2.6) B.1.351 (4.9) B.1.617.2 (5.8)	[34]
mRNA-1273 (8)	7-days	Vike mutations of variants (ID_50_)	Wuhan-1 isolate with D614G exchange (reference) B.1.1SV pseudotyped with respective sp.7 (1.2) P.1 (3.2) B.1.351 (6.9 ~ 8.4) B.1.617.2 ( 2.1)	[35]
ChAdOx1 (49)	28-days	Authentic virus microplate neutralization (ND_50_)	Victoria (reference) B.1.1.7 (9)	[37]
CoronaVac (60)	15-days (approx.)	Authentic SARS-CoV-2 microplate neutralization (GMT)	Prototypic vaccine strain (wild-type) (reference) B.1.17 (17.35) B.1.351 (22.11) B.1.617.2 (31.64)	[38]
BNT162b2 (180)	3-weeks	Authentic virus microneeutrilization test (MNT titer)	B.1 and B.1.1.7 (reference) B.1.351 (5)	[39]
mRNA-1273 (12)	15-days	Authentic SARS-CoV-2 microplate neutralization (ID_50_) and Pseudovirus neutralization assays (ID_50_)	WA1/2020 (reference) B.1.1.7 (essentially unchanged) B.1.351 (12.4 and 8.6)	[40]
BNT162b2 (10)	≥ 7-days		WA1 (reference) B.1.1.7 (essentially unchanged) B.1.351 (10.3 and 6.5)	
BNT162b2	2-4-weeks	Mutant viruse’s spike engineerd into USA-WA1/2020	WA1/2020 (reference) B.1.17 (0.8) P1 (1.2)	[41]
Ad26.COV2.S (20)	^b^71-days post vaccination	Pseudovirus-based neutralization assay. (Median pVNA titer)	WA1/2020 (reference) B.1.351 (5.0) P.1 (3.3)	[42]
		Authentic virus neutralization assay. (Median live virus neutralizing antibody titer)	WA1/2020 (reference) B.1.351 (10.6)	
BNT162b2 (W1, n = 10) W3, n = 15)	1-week 3-weeks	S-Fuse neutralization assay using authentic virus (ED_50_)	hCoV-19/France/GE1973/2020, B.1.17 (reference) ^a^ B.1.351 (14 and 53)	[43]
BNT162b2 (15)	24 to 31-days	Pseudotyped VSV particles with the spike (NT_50_)	Wuhan-1 isolate with D614G exchange (reference) B.1.351 (11.13)	[44]
BNT162b2 (20)	2 to 4-weeks	Authentic infectious virus (PRNT_50_)	WA1/2020 (reference) B.1.617.2 (1.41)	[45]
mRNA-1273 (15)	35 to 51-days	Authentic virus (FRNT_50_)	WA1/2020 (reference) B.1.617.2 (3)	[46]
BNT162b2 (10)	7 to 27-days		WA1/2020 (reference) B.1.617.2 (3.3)	

^a^14-fold and 53-fold lower against B.1.351, when compared to D614G and B.1.117, respectively, 4-weeks post second shot. ^b^ single dose. ED_50_ = effective dose 50 .GMT = geometric mean titer. IC_50_ = inhibitory concentration 50. ID_50_ = inhibitory dilution 50. ND_50_ = neutralization dose 50. NT_50_ = neutralization titer 50pVNA = pseudovirus neutralizing assay. PRNT_50_ = 50% plaque reduction neutralization testing. FRNT_50_ = 50% focus reduction neutralization test VSV = Vesicular stomatitis virus. W1 = 1- week post second dose and W3 = 3-weeks post second dose.

**Table 3:** Immune response to heterologous vaccination.

Prime (n)	Booster (n)	Time interval (weeks)	^a^Sample collection	Assays for antibody titration	Assays for T cell responses	Weakness of study	Reference
ChAdOx1	BNT162b2 (450)	8–12 weeks	14-days	pVNA Commercial immunoassays	ELISA to quantify IFN-γ in overnight stimulated heparinized whole blood stimulated with pools of SARS-CoV-2 spike peptides.	No ChAdOx1 prime boost group. No mRNA vaccine prime and ChAdOx1 booster group.	[61]
ChAdOx1 (222)	NA						
ChAdOx1	ChAdOx1 (17)	^b^82	0 to 3 days before and 19 to 21 days after boost vaccination	Chemiluminescent immunoassay Bead-Based Multiplex assay Surrogate virus neutralization test	__	No BNT prime ChAd booster group. Different prime boost intervals between groups.	[63]
ChAdOx1	BNT162b2 (159)	83					
BNT	BNT162b2 (159)	20					
ChAdOx1	ChAdOx1 (36)	10–12 Weeks	20–28 days 20–21 days 27–31 days	Microarray-based immunoassay Surrogate SARS-CoV-2 virus neutralization test. pVNA	ELISA to quantify IFN-γ in heparinized whole blood stimulated with pools of SARS-CoV-2 S1 peptides.	ChAdOx1 prime boost group comparatively smaller. Different intervals between prime and boost vaccination.	[66]
ChAdOx1	BNT162b2 (104)	10–12 Weeks					
BNT162b2	BNT162b2 (159)	3 weeks					
ChAdOx1	ChAdOx1 (32)	73 days	16 days 17 days	Spike IgG and IgA, and reciprocal titers of neutralizing antibodies	Flow cytometry to analyze total number of spike-specific cytokine secreting CD4^+^ and CD8^+^ T cells. PBMCs were stimulated for 12-16 hours	No BNT prime booster, BNT prime, ChAd booster groups	[67]
ChAdOx1	BNT162b2 (159)	74 days					
ChAdOx1 (222)	NA						
ChAdOx1	ChAdOx1 (55)	9-12	9-12 weeks	ELISA Neutralization assay	Flow cytometry to analyze S-specific CD4^+^ and CD8^+^ T cells. Heparinized whole blood was stimulated by S overlapping peptides for 6 hours in presence of co-stimulatory antibodies against CD28 and CD49d.	No mRNA vaccine prime and ChAd group	[68]
^c^ mRNA vaccine	mRNA vaccine (64)	3-6	3-6 weeks				
ChAdOx1	mRNA vaccine (97)	9-12	9-12 weeks				
ChAdOx1	BNT162b2 (2)	33 days	13 days	ELISA pVNA	__	N is very small and no ChAdOx1 prime-boost, BNT162b2 prime boost, and BNT162b2 prime covishield booster groups.	[69]
ChAdOx1	ChAdOx1 (25)		28 days	Authentic virus neutralization assay pVNA	IFN-γ ELISpot specific to SARS-CoV-2 spike peptides, analyzed on PBMCs		[71]
ChAdOx1	BNT162b2 (24)						
BNT162b2	BNT162b2 (26)						
BNT162b2	ChAdOx1 (25)						

^a^Time points are related to evaluation of humoral or cellular immune responses post second dose. ChAdOx1 represents ChAdOx1 nCoV-19. NA = not applicable. ^b^median (interquartile range, IQR) days. ^c^mRNA-vaccinees included either BNT162b2 or mRNA-1273 vaccinees. pVNA = pseudovirus neutralization assay. PBMCs = peripheral blood mononuclear cells, S = spike protein.
